# The Effect of Preoperative Hematocrit Levels on Early Outcomes After Coronary Artery Bypass Graft

**DOI:** 10.7759/cureus.12733

**Published:** 2021-01-16

**Authors:** Suresh Kumar, Naresh Kumar Khurana, Inayatullah Awan, Sidra Memon, Muhammad Khizar Memon, Hamza Sohail, Basma Ali, Besham Kumar

**Affiliations:** 1 Internal Medicine, Bolan University of Medical and Health Sciences, Quetta, PAK; 2 Cardiology, Central Park Medical College, Lahore, PAK; 3 Psychiatry, Ghulam Muhammad Mahar Medical College, Sukkur, PAK; 4 Internal Medicine, Jinnah Sindh Medical University, Karachi, PAK; 5 Internal Medicine, Liaquat University of Medical and Health Sciences, Jamshoro, PAK; 6 Internal Medicine, Jinnah Postgraduate Medical Centre, Karachi, PAK

**Keywords:** myocardial infarction, hematocrit, cabg

## Abstract

Introduction

Coronary artery bypass graft (CABG) is the most potent of surgical procedures; in this procedure, the narrowing of the coronary artery due to atherosclerotic plaque is bypassed by forming an alternate route for blood flow to the heart. There are various risk factors associated with the procedure. The aim of this study was to observe if postoperative outcomes are affected by preoperative hematocrit (hct) levels in patients.

Methods

This longitudinal study was conducted from April 2019 to December 2019. Eighty-two (82) participants who were to undergo CABG surgery were divided into two groups based on their preoperative hct levels. Group 1 had 42 participants with lower levels of hct (less than 35.5% for women and 38.3% for men), whereas group 2 consisted of 40 participants with normal hct levels (greater than 35.5% for women and 38.3% for men).

Results

The results showed that participants undergoing CABG with lower than normal hct levels had increased blood loss through drainage as compared to participants who had normal hct levels (680.1 ± 301 mL vs. 500.7 ± 412 mL; p-value: 0.02). Group 1 participants also had an increased need for blood and blood product transfusion as compared to group 2 (3.2 ± 1.8 units vs. 1.8 ± 0.9 units; p-value: <0.0001). Furthermore, the participants in group 1 had longer stays in the ICU relative to the other group (5.2 ± 3.1 days vs. 3.4 ± 2.5 days; p-value: 0.003).

Conclusion

Based on our findings, patients who undergo CABG surgery with lower than normal hct levels are at increased risk of certain complications, including excessive blood loss, need for transfusion, and increased duration of ICU stay. Therefore, preoperative hct levels should be routinely checked in patients undergoing CABG to prevent these complications.

## Introduction

Coronary artery bypass graft (CABG) is a revascularization procedure that is used to treat coronary artery disease (CAD), which is the narrowing of the arteries supplying oxygen and nutrients to the myocardium by atherosclerotic plaques [1]. Considered as the most effective treatment for ischemic heart disease (IHD), CABG improves blood flow to the heart by connecting a piece of a healthy blood vessel from elsewhere in patients’ body, most commonly the internal mammary artery, to positions above and below the narrowed or blocked portion of the coronary artery, thus bypassing the obstruction [1]. Patients with high-risk EuroSCOREs or those with complex atherosclerotic lesions, multivessel disease, left main coronary artery disease (LMCA), left ventricular dysfunction (LVD), and diabetes mellitus (DM) derive more benefits from CABG than from percutaneous coronary intervention (PCI), which is another revascularization procedure performed for the treatment of CAD [2].

Various studies have been conducted in the past to determine the surgical risk factors of CABG, and it has been found that the success of the procedure depends on the improvement or elimination of these risk factors, making preoperative risk-factor assessment important in patients undergoing CABG [3-5]. These risk factors include advanced age (older than 65 years), ventricular dysfunction, complex or diffuse coronary lesions, previous cardiac surgery, complicated surgery, and poor respiratory or renal function [3]. A thorough preoperative assessment through clinical history, physical examination, and investigations such as full blood count including hematocrit (hct) levels, patient’s coagulation profile, blood group determination, measurement of serum electrolytes, urea, creatinine and hepatic enzymes, a 12-lead ECG, chest X-ray, and a left heart catheter can improve postoperative outcomes in patients undergoing CABG [6,7].

There is very limited data available on the preoperative assessment of patients undergoing CABG in the local setting. In this study, we aimed to analyze the effect of hct levels on early postoperative outcomes in patients undergoing CABG.

## Materials and methods

This longitudinal study was conducted in the cardiovascular unit of a tertiary care hospital in Pakistan from April 2019 to December 2019. The sample size was calculated using an online calculator, ClinCalc (ClinCalc LLC; https://clincalc.com/stats/samplesize.aspx) based on results from Pala et al. [3]. After adjusting for patients lost to follow-up during the study period, the sample size was determined to be 82. These participants had undergone CABG after providing informed consent. The inclusion criteria included patients with silent, stable, or unstable angina and presence of at least two lesions in different coronary arteries, and thus eligible for revascularization via CABG. Patients with a previous history of CABG surgery, patients who had to undergo emergency surgery due to hemodynamic instability, and patients with chronic kidney diseases were excluded from the study as these conditions may influence the outcome of the procedure.

Demographic data such as age, gender, hypertension, and type 2 diabetes status were recorded in a self-structured questionnaire. The ejection fraction was noted via transthoracic echocardiography a day before the procedure. During the procedure, the length of mechanical ventilation, drainage, and blood transfusion was noted. After the procedure, the length of ICU stay and the length of hospital stay were noted. Patients were followed up for 30 days for mortality.

Statistical analysis was performed using SPPS Statistics version 23.0 (IBM, Armonk, NY). Continuous variables were presented as means and standard deviations. Binary outcomes were expressed as frequencies and percentages. The student's t-test and Pearson's chi-squared test were employed to compare the two groups. A p-value of less than 0.05 indicated that there was a significant difference between the groups and the null hypothesis was not valid.

## Results

After enrollment in the study, all patients underwent phlebotomy before any intervention was given, and blood samples were sent to the laboratory to determine the hct levels. Forty-two (51.3%) patients undergoing CABG had lower levels of preoperative hct (less than 35.5% for women and 38.3% for men) and were placed in one group (group 1), and the other 40 (58.7%) participants with normal hct levels (greater than 35.5% for women and 38.3% for men) were placed in another group (group 2).

Age, gender distribution, comorbidities (hypertension and type 2 diabetes) and ejection fraction were comparable between both groups. Hemoglobin (Hb) and hct were significantly lower in group 1 compared to group 2 (Table 1).

**Table 1:** Demographic data of the participants *Statistically significant SD: standard deviation; Hb: hemoglobin; hct: hematocrit; n: number; g/dL: grams/deciliter; NS: non-significant

Variables	Group 1 (n=42)	Group 2 (n=40)	P-value
Age in years, mean ± SD	62 ± 8	59 ± 9	NS
Males (%)	78.5	70.0	NS
Hypertension (%)	82.5	85.7	NS
Type 2 diabetes (%)	59.5	55	NS
Ejection fraction (%), mean ± SD	42.3 ± 9.2	44.4 ± 8.7	NS
Preoperative Hb (g/dL), mean ± SD	9.9 ± 1.1	13.1 ± 1.4	<0.0001*
Preoperative hct (%), mean ± SD	31.8 ± 2.5	39.9 ± 2.8	<0.0001*

Results showed that participants undergoing CABG with lower than normal hct levels had increased blood loss through drainage as compared to participants who had normal hct levels (680.1 ± 301 mL vs. 500.7 ± 412 mL; p-value: 0.02). Group 1 participants also had an increased need for blood and blood product transfusion as compared to group 2 (3.2 ± 1.8 units vs. 1.8 ± 0.9 units; p-value: <0.0001). Furthermore, the participants in group 1 had longer stays in the ICU relative to the other group (5.2 ± 3.1 days vs. 3.4 ± 2.5 days; p-value: 0.003) (Table 2).

**Table 2:** Comparison of outcomes between group A and group B *Statistically significant SD: standard deviation; n: number; cc: cubic centimeter

Outcome	Group 1 (n=42)	Group 2 (n=42)	P-value
Mechanical ventilation duration (hours), mean ± SD	13.2 ± 7.2	12.1 ± 5.1	0.46
Drainage amount (cc), mean ± SD	680.1 ± 301	500.7 ± 412	0.02*
Blood and blood product transfusion (unit), mean ± SD	3.2 ± 1.8	1.8 ± 0.9	<0.0001*
Reoperation for bleeding, n (%)	7 (17.5%)	4 (9.5%)	0.28
Length of stay in the ICU (days), mean ± SD	5.2 ± 3.1	3.4 ± 2.5	0.003*
Length of hospital stay (days), mean ± SD	17.2 ± 10.6	14.9 ± 5.6	0.21
Mortality (in 30 days), n (%)	4 (10%)	2 (4.76%)	0.36

## Discussion

Patient optimization prior to CABG is essential for better outcomes and early recovery. Hematocrit is a well-recognized factor with respect to pre and postoperative complications of cardiac surgery. The present study was conducted to assess the effect of hct levels on early adverse postoperative outcomes in patients undergoing CABG. In our study, postoperative drainage, the need for blood and blood product transfusion, and length of ICU stay were found to be statistically higher in patients in the low-hct group.

Despite the advances in cardiovascular medicine and cardiac surgery operating techniques, morbidity and mortality are still a problem after CABG [8]. With increasing life expectancy, morbidity and mortality rates have also increased in elderly individuals. Due to this reason, preoperative assessment in this age group is extremely important to determine the risk factors. By using risk stratification scoring systems, operating surgeons, cardiac anesthetists, ICU teams, patients' relatives, and the patients themselves become aware of what awaits them. Moreover, awareness of these factors could enable appropriate preoperative treatments to be provided, thereby reducing the risk for patients [9]. The assessment is also very important to determine preoperative risk because it increases the complications after surgery, which ultimately increases the cost and prolongs the hospital stay [10,11].

Multiple studies have been conducted about this topic, and they have investigated the association of blood and blood product transfusions with outcomes in CABG patients. In a study by Surgenor et al., 36% of patients received one to two units of packed cell transfusion; among them, 43% were transfused intraoperatively, and the rest were postoperatively transfused, and the mortality rate was found to be 16% higher in patients receiving transfusion compared to those who did not receive it [12]. A study conducted at the Cleveland Clinic on 15,000 patients to assess the correlation between transfusion and postoperative infections showed that transfusion was associated with increased incidences of bacteremia and superficial and deep wound infections [13]. In the present study, patients in group 1 received more transfusions and had prolonged hospitalization and ICU stay compared to group 2; both findings were statistically significant (p: ≤0.005). These findings are consistent with a study conducted in the UK, which reported prolonged hospital and ICU stay for patients who received transfusion compared to those who had no transfusion history or received less than 1 transfusion unit [14].

A low preoperative hct level is an independent risk factor for the need for a transfusion, and the mortality rate among patients is directly proportional to the need for a transfusion [15]. Similarly, several studies have shown that low preoperative hct levels in patients undergoing cardiac surgery triggered postoperative complications [16,17]. Ranucci et al. found that patients with preoperative hct levels of ≤33% developed more than three-fold levels of major morbidities when compared to patients with preoperative hct levels of ≥42% [18].

In the current study, the mortality rate was higher in group 1 compared to group 2, although the difference was statistically insignificant. Low preoperative hct levels have a negative impact on postoperative recovery, and the higher initial 30-day mortality rate in this patient group is an indication of the need for careful treatment planning. Even if the risk scoring system did not include the hct level, attention should be paid to the hct test as it will not only help the surgeon but also the patient in terms of decision-making and planning regarding the operation.

The major limitation of the current study was that it was conducted at a single center; therefore, our results cannot be generalized to other centers with a higher number of patients. Another limitation was that patients were analyzed for in-hospital mortality only and were not followed up for medium and long-term results.

## Conclusions

In the present study, low preoperative hct levels were found to be associated with prolonged ICU stay among CABG patients. It was also associated with an increased need for blood transfusion and blood drainage during and after CABG, which are possible determinants of various adverse events. Therefore, detecting and treating low preoperative hct levels is critical as it may reduce unwanted postoperative complications. Moreover, we believe that preoperative hct levels should be an integral component of risk scoring systems to assess the postoperative mortality risk and to foresee hospital stay and cost.
